# The Influence of Financial Development on Energy Consumption: Worldwide Evidence

**DOI:** 10.3390/ijerph17041428

**Published:** 2020-02-23

**Authors:** Xiaoxin Ma, Qiang Fu

**Affiliations:** 1 Economics and Management School, Wuhan University, Wuhan 430072, China; 2 Postdoctoral Workstation of China Construction Bank, Beijing 100032, China; 3 Postdoctoral Research Station of Renmin University of China, Beijing 100872, China

**Keywords:** financial development, energy consumption, worldwide evidence, system GMM

## Abstract

In this study, we investigated the influence of overall financial development and its components on energy consumption using the panel data of 120 countries and the generalized method of moments (GMM). By dividing the sample into developed and developing countries, we further examined the national differences of the impact of financial development on energy consumption. The empirical results indicate that the overall financial development significantly positively impacts energy consumption from a worldwide perspective, and its two components (financial institution and the financial market) have the same effect. The analysis of national differences indicates that the financial development also positively impacts energy consumption in developing countries but with no obvious effect in developed countries. The results also suggest that financial development cannot be used to restrain the increase in energy consumption from the global perspective, and policymakers in developing countries must balance the relationship between the development of the financial sector and energy consumption.

## 1. Introduction and Literature Review

Energy is one of the key elements in producing most goods and services and is essential for promoting economic growth [1,2,3]. However, the rapid increase in energy consumption has placed tremendous pressure on the environment. Many empirical studies have reported that energy consumption has a significantly positive relationship with carbon emissions or the discharge of other pollutants; consequently, scholars found that energy consumption is an important factor leading to environmental degradation [4,5,6]. Future energy supply is becoming a serious issue. Although many countries have attached importance to the development of different types of new energy, due to various restrictions of their application, fossil fuels such as coal and oil still occupy the main share of the world’s energy structure at present. However, as the traditional fossil fuels are non-renewable and will be exhausted in the next few decades, most countries will face enormous energy supply challenges. The ever-increasing population is further aggravating this situation.

Accordingly, analyzing the factors influencing energy consumption could help authorities to formulate energy policies and balance the relationship among economic growth, energy supply, and environmental protection.

The common thought is that some conventional variables, such as trade openness, urbanization, and industrialization, affect energy consumption [7,8,9]; however, more recently, many scholars have proposed that, as an important component of macro economy, financial development is a non-negligible factor that could significantly influence energy consumption. The financial development generally refers to the scale expansion and efficiency improvement of the domestic financial sectors. A series of researchers have examined the relationship between financial development and energy consumption from both theoretical and empirical perspectives.

Theoretically, many scholars [10,11,12] have considered that financial development increases energy consumption because a well-developed financial system could provide funds for enterprises with much lower costs, which facilitates the expansion of their production scale and therefore increases energy consumption. Financial development also increases consumer access to consumption credit, dramatically encouraging them to purchase more commodities, including automobiles and electric appliances, which further stimulates energy demand. However, other scholars [13,14,15] proposed that financial development reduces energy consumption because a developed financial system can mitigate the financial constraints on enterprises to help them update production technologies and equipment, which could effectively improve energy efficiency. Financial development could also help enterprises increase their research and development (R & D) investment, and design and manufacture of advanced energy-saving products, thereby reducing aggregate energy consumption.

Various empirical studies have explored the relationship between financial development and energy consumption with different samples and methodologies. However, these studies provided widely different conclusions, which have not reached a consensus.

Firstly, financial development was found to increase energy consumption. Sadorsky [10] analyzed the relationship between financial development and energy consumption based on generalized method of moments (GMM) estimation techniques and the data of 22 emerging countries from 1990 to 2006 and concluded that financial development has a positive and significant impact on energy consumption when financial development is measured using three stock market variables. Based on the panel data of nine central and eastern European frontier countries, Sadorsky [16] examined the influence of the banking sector and stock market on energy consumption. The empirical results indicated that when using three banking variables, a significantly positive relationship exists between financial development and energy consumption; however, when using three stock market variables, only stock market turnover has a significantly positive relationship with energy consumption. Al-mulali and Lee [17] investigated the effect of financial development on energy consumption in Gulf Cooperation Council (GCC) countries using the Pedroni cointegration test and panel data for 1980–2009. The results showed that financial development is an important factor promoting the increase in energy consumption in both the short and long term. Ahmed [18] studied the nexus of financial development and energy consumption using a series of panel data methodologies and BRICS (Brazil, Russia, India, China, and South Africa) data from 1991 to 2013 and concluded that financial development increases energy intensity in BRICS countries, despite sustainable development measures being adopted. Mukhtarov et al. [19] examined the impact of financial development on energy consumption based on cointegration, a vector error correction modeling framework, and the annual data of Azerbaijan from 1992 to 2015. They discovered that financial development significantly positively impacts energy consumption in the long term: A 1% increase in financial development produces a 0.12% increase in energy consumption.

Secondly, financial development has also been found to reduce energy consumption. Farhani and Solarin [20] investigated the relationship between financial development and energy demand with a newly developed LM unit root test and quarterly data of the United States from Q1 1973 to Q4 2014 and concluded that although financial development stimulates energy demand in the short term, energy demand decreases in the long term. Destek [21] examined the influence of financial development on energy consumption using the common correlated effect (CCE) estimator and annual data from 1991 to 2015. The empirical results indicated that the development of the banking and bond markets has a significantly negative effect on energy consumption. Ouyang and Li [22] analyzed the effect of financial development on energy consumption based on a GMM panel VAR (Vector Autoregressive Regression) approach using the quarterly panel data of 30 Chinese provinces from Q1 1996 to Q4 2015. Financial development was measured by a series of indicators including a comprehensive indicator that was calculated using principal component analysis (PCA). The results showed that financial development could significantly lessen energy consumption. Through full-sample analysis by dividing the sample into three sub-samples (eastern, central, and western China), the authors further analyzed the regional differences and found that the inhibitory effect of financial development on energy consumption was largest in the western region, followed by the eastern region and then the central region. Gomez and Rodriguez [3] researched the influence of financial development on energy consumption in North American Free Trade Agreement (NAFTA) countries using a series of panel data methods and panel data from 1971 to 2015 and found a negative relationship between financial development and energy consumption.

Thirdly, other studies reported no significant correlation between financial development and energy consumption. Coban and Topcu [23] examined the nexus of financial development and energy consumption in the European Union (EU) using system GMM model and panel data from 1990 to 2011. The results indicated that financial development had a positive relationship with energy consumption in the old members of the EU; however, this relationship between financial development and energy consumption was not significant in the EU27. Based on the Johansen co-integration test and VAR Granger causality, using data covering the period from 1960 to 2011 in Turkey, Keskingoz and Inancli [24] found that although bank deposits have a positive relationship with energy consumption in the short term, no obvious correlation exists between financial development and energy consumption in the long term. Topcu and Payne [25] investigated the relationship between financial development and energy consumption with two heterogeneous estimation approaches, mean group (MG) and common correlated effect mean group (CCEMG), and the panel data of 32 high-income countries from 1990 to 2014. They constructed an overall index and one index each for the banking sector, stock market, and bond market to measure financial development and found that increases in the stock market index cause slight declines in energy consumption; however, the overall index had no statistical relationship with energy consumption. 

Lastly, in another group of studies, a nonlinear relationship was found between financial development and energy consumption. Baloch et al. [26] examined the influence of financial development on energy consumption in Organization for Economic Co-operation and Development (OECD) countries with the Driscoll–Kraay standard errors panel regression model using panel data covering the period from 1980 to 2016. They concluded that an inverted U-shape relationship exists between financial development and energy consumption. Yue et al. [27] studied the effect of financial development on energy consumption based on the panel smooth transition regression (PSTR) model and the panel data of 21 transitional countries from 2006 to 2015. The empirical results showed that no obvious linear relationship exists between financial development and energy consumption where the nonlinear parameters are significant. Specifically, the development of financial intermediation increases energy consumption in all countries, the development of the stock market reduces energy consumption in China and Poland, and the development of financial openness negatively affects energy consumption except in Georgia and the Kygyz Republic. Sare [28] investigated the relationship between financial development and energy consumption using a sample splitting and threshold estimation approach and the panel data of 45 African countries covering the period from 1973 to 2017. The results showed that the impact of financial development on energy consumption has a threshold effect. Specifically, financial development increases energy consumption when the index of financial development is below the threshold; however, this promotion effect gradually weakens and eventually transitions to an inhibitory effect when the index of financial development is above the threshold.

The theoretical analysis indicates that the financial development has two opposite impacts on energy consumption, which results in the fact that the aggregate impact of financial development on energy consumption may be difficult to be identified. In fact, the empirical analysis strongly supports the viewpoint of theoretical analysis, as the empirical analysis with different samples and methodologies provide widely different conclusions, which indicate that the impact of financial development on energy consumption varies across countries. 

These circumstances reflect the complexity of the relationship between financial development and energy consumption, which we should discreetly regard. Although the relevant literature cannot reach a unique conclusion, they have provided us a comprehensive view of the influence of financial development on energy consumption across different countries which could facilitate energy policy making.

Nevertheless, it is notable that most of the empirical research concentrated on specific countries or regions, few studies focused on this topic from the worldwide perspective. Generally, the regional research could provide the policymakers the concrete conclusions and suggestions concerning corresponding countries or regions; however, they are unable to reflect the universality of the impact of financial development on energy consumption. Conversely, although the research from worldwide perspective neglects the characteristics of different countries, it could provide us a “aggregate” view on this topic, which could assist with relevant energy and environmental policy making. In other words, it reflects the relationship between financial development and energy consumption on a macro level, which acts as a benchmark. Therefore, in this study, we analyzed the influence of financial development on energy consumption from a global view to provide macro empirical evidence on their relationship.

Specifically, there are three primary purposes of this study: (1) Analyze the comprehensive influence of financial development on energy consumption based on the panel data of 120 countries, which provides us a macro perspective on their relationship. (2) Investigate the effect of different components of financial development on energy consumption by dividing the financial development into financial institution and financial market. Due to the rich connotations of financial development, it is necessary to check whether its different components have distinct impact on energy consumption. (3) Considering the discrepancy, such as economic structure and technical level, which different types of countries may have, we further examine the national difference of the relationship between financial development and energy consumption by dividing the sample into developed and developing countries.

The remainder of the paper is arranged as follows: Section 2 introduces the methodology and data, Section 3 presents the empirical results, Section 4 describes the robustness checks, and Section 5 concludes this paper and outlines policy implications.

## 2. Empirical Strategy and Data Selection

### 2.1. Model and Methodology

The main objective of this paper is to analyze the influence of financial development on energy consumption from worldwide perspective. To realize this objective, we adopt dynamic panel model for analysis. Compared with the static panel model, the dynamic panel model added a lag term of the explained variable as the explanatory variable. This could reflect the dynamic process of energy consumption, which fits the reality. Furthermore, adding a lag term could also improve the credibility of regression results by relieving the influence of uncontrollable factors. The dynamic panel model we built is as follows:

*EC_it_* = *α* + *β*_0_*EC*_*it*–1_ + *β*_1_*FD_it_* + *γControl_it_* + *μ_i_* + *ε_it_* (1)

where *EC_it_* denotes energy consumption; *FD_it_* represents financial development; *Control_it_* indicates the control variables that may affect energy consumption; *β*_0_, *β*_1_, and *γ* denote coefficients of the corresponding explanatory variables; *μ_i_* is the unobserved country-specific effect; *ε_it_* is the residual term; and *i* and *t* denote country and year, respectively. *EC_it_*_–1_ is the lag term of energy consumption.

Due to the existence of the lag term of the explained variable, we could not estimate the above model with traditional methods, such as fixed or random effects, due to the existence of an endogenous problem; consequently, the effective estimators could not be obtained. Therefore, the GMM (generalized method of moments) [29,30,31] was adopted to estimate the results. Compared with other estimation methodologies, GMM could effectively deal with the problems of endogeneity and omitted variable biases. GMM consists of difference GMM and system GMM, and according to the selection of different weight matrixes, each of them can be divided into one-step and two-step GMM. Generally, the system GMM performs better in improving the efficiency of estimation than difference GMM, and the two-step GMM performs better in handling the heteroscedasticity and autocorrelation compared with one-step GMM. Therefore, we adopted a two-step system GMM for estimation.

Stata software (StataCorp, College Station, TX, USA) and the command “xtabond2” were used to conduct the estimations. Roodman [32] provided more information about this command. The test of serial correlation was used to confirm the consistency of the estimator, based on the statistics of the first- and second-order autocorrelation estimators. We adopted the Hansen test to judge the effectiveness of the instrument variables. We selected the Hansen test rather than the Sargan test because the Sargan test is not robust with respect to heteroscedasticity or autocorrelation [32].

### 2.2. Data Selection

Currently, no indicator is widely accepted as the most suitable proxy of financial development due to its complex connotation. Various indexes have been adopted by scholars based on their research objectives and data availability. Earlier studies mostly used a single proxy variable; scholars now generally adopt a series of indictors or construct a comprehensive indicator based on several existing indexes and relevant statistical techniques to measure financial development [33,34,35], as the empirical results may depend on how financial development is measured [23,33].

As such, in this study, we used the comprehensive index proposed by Svirydzenka [36] as the proxy variable of financial development. Given the connotation and theories of financial development, Svirydzenka [36] divided the index into financial institution and financial market and further measured each from three aspects: Depth, access, and efficiency. Table 1 presents the framework of this index.

Svirydzenka [36] first calculated six second-level sub-indexes (FID, FIA, FIE and FMD, FMA, FME) with a series of original variables. For instance, the depth of financial institutions is calculated using four variables: Private sector credit to gross domestic product (GDP), pension fund assets to GDP, mutual fund assets to GDP and insurance premiums, and life and non-life to GDP. Using these six indexes, the author then calculated two first-level sub-indexes, FI and FM, to indicate the development of the financial institution and financial market. Lastly, the comprehensive index, FD, was calculated based on FI and FM to measure the overall financial development. The three advantages of this index are as follows: first, this index is constructed based on 20 original variables, including many variables that are commonly adopted in empirical research that can reflect the rich connotation of financial development to the greatest extent. Second, various statistical methodologies are adopted in the process of index construction, and treatment of missing data has been carefully considered, increasing the reliability of the results. Third, the dataset covers more than 180 countries for the period of 1980 onwards, which allowed us to significantly expand our sample size. Consequently, we selected this index to measure overall financial development. We used two first-level sub-indexes, financial institution (FI) and financial market (FM), to analyze the effect of different components of financial development on energy consumption. Please refer to Svirydzenka [36] for more detailed information concerning these indexes.

We also adopted the domestic credit to the private sector (% of GDP) index as the proxy variable of overall financial development in the robustness checks to guarantee the reliability of the empirical results. This index has been deemed one of the most suitable indexes among the single indicators to measure financial development and has been widely used in the literature [38,39,40].

Following common practice in the research of energy consumption [10,18,41], we adopted energy use (kg of oil equivalent per capita) as its proxy variable and added three control variables in the model: Trade openness, urbanization, and industrial structure. Table 2 presents the variable descriptions of our model.

Generally, scholars wish to expand their samples as much as possible while conducting empirical research to strengthen the reliability of the regression results. However, a negative correlation exists between the amount of sample and the period for panel data which the researchers should carefully balance to obtain the maximum observations. For the variables we selected, we found that the data initiated from 1991 and ended in 2014 could maximize our dataset (especially for the data of energy consumption), the expansion of sample period will cause the huge loss of sample countries. Therefore, we included 120 countries covering the period from 1991 to 2014 in the main regression. Table A1 in Appendix A presents the specific sample countries.

As a series of regressions are presented in this paper, the exact sample size and period will be presented in the corresponding tables for each regression. To analyze the national difference of the relationship between financial development and energy consumption, we divided the sample countries into developed and developing countries based on the country classification of the International Monetary Fund (IMF) [42] for a total of 34 developed countries and 86 developing countries in the main regression. The FD1, FI, and FM variables were proposed by Svirydzenka [36], and all other variables were extracted from the World Development Indicators database of the World Bank.

The energy consumption (EC) variable was transformed into the natural logarithm to mitigate the issues of nonnormality and heteroscedasticity [43]. We did not conduct this transformation for other variables, as they are dimensionless or ratio indexes. Table 3 presents the descriptive statistics of the main regression.

From Table 3, we can notice that the statistics of all variables are in the normal range, which means the model will not be disturbed by extreme values. It is necessary to explain that although there is a large gap between the minimum and maximum of EC, this reflects the remarkable difference of energy consumption across countries, but is not caused by extreme values, as the data of EC expresses the state of continuous change in the range between minimum and maximum.

For the purpose of simplicity, we use the word “country” throughout this paper to refer to “country and region”. Similarly, according to the IMF country classification [42], the sample countries were divided into “developed countries” and “emerging market and developing countries”, for which we use “developing countries” for the latter. We conducted a series of regressions to comprehensively analyze the influence of financial development on energy consumption; to save space and avoid confusion, we only report the descriptive statistics and the results of the unit root tests and the co-integration tests for the main regression (full sample). Those of the other regressions are available upon request.

## 3. Results and Comments

### 3.1. Unit Root Test and Co-Integration Test

Before the empirical analysis, we first conducted unit root tests to examine the stationarity of the data, as non-stationary data may lead to the issue of spurious regression. To guarantee the reliability of the results for the unit root tests, five commonly-used methodologies [44,45,46,47,48] were used to examine the stationarity of both the original values and the first-order difference of the variables. Table 4 presents the results of the unit root tests, indicating that the original values of all variables passed the stationary tests. We further adopted three co-integration tests [49,50,51] to examine the co-integration relationship among these variables, and the results in Table 5 indicate, all three tests rejected the null hypotheses of no co-integration, implying that these variables have a long-term co-integration relationship.

We also used the unit root tests and co-integration tests for all other regressions in this paper and all passed these tests. This indicates that the data we selected were suitable for analysis based on panel data methodologies.

### 3.2. Effect of Overall Financial Development on Energy Consumption

First, we examined the influence of overall financial development on energy consumption from the worldwide perspective using FD1 as the proxy variable of financial development. We gradually added the control variables to the regression to verify the robustness of the effect of financial development. Table 6 presents the empirical results.

Table 6 shows that for all regressions, the coefficients of FD1 were positive and significant at least at the 5% level. The statistics for AR (1) were significant at the 1% level, whereas those for AR (2) were insignificant, which indicated that the results were not affected by second-order autocorrelation. The statistics of the Hansen test were insignificant, implying that the null hypothesis of instrumental variables being jointly valid could not be rejected, verifying that the selection of instrumental variables in the model was appropriate. The empirical results implied that overall financial development has a positive effect on energy consumption; the stepwise regressions and the misspecification tests proved the reliability of this conclusion.

The theoretical analysis indicated that the financial development has both a promoting effect and an inhibitory effect on energy consumption. Financial development increases energy consumption by mitigating the financial constraints on enterprises and providing more consumption credit to consumers, which facilitates the expansion of the enterprise production scale and encourages the consumers to purchase more commodities, respectively. Conversely, financial development reduces energy consumption as it supports enterprises to improve energy efficiency and manufacture advanced energy-saving products by updating production technologies and equipment and by increasing the amount of R & D investment. Therefore, the above results indicate that, comprehensively, the promoting effect exceeded the inhibitory effect and dominated in our sample countries. We conclude that the overall financial development has a positive impact on energy consumption from a global perspective. This conclusion is consistent with the works of Sadorsky [10], Al-mulali and Lee [17], and Mukhtarov et al. [19].

### 3.3. Effect of Different Components of Financial Development on Energy Consumption

The previous section examined the influence of financial development on energy consumption from a comprehensive perspective using the FD1 index. Due to the various connotations of financial development, scholars believe it is necessary to conduct the analysis based on the components of financial development, which may result in further discoveries and implications. Many scholars have investigated the relationship between financial development and energy consumption from different aspects of financial development. Therefore, we analyzed the impact of different components of financial development on energy consumption to provide more global evidence of their relationship.

Following general practice [39,52], we divided the financial development into two components: financial institutions and the financial market, which correspond to the forms of indirect financing and direct financing, respectively. Financial institutions, including mainly banks, generally provide long-term loans that require collateral and strict inspection. Conversely, the financial market, such as the stock market, can allow enterprises to raise funds directly from investors at relatively low costs. The FI and FM indexes proposed by Svirydzenka [36] were used to measure the development of financial institutions and the financial market, respectively. Table 7 presents the regression results.

According to the empirical results, the coefficients of FI and FM were 0.0706 and 0.0463, respectively. Both were positive and significant at the 1% level, which is consistent with the results using the FD1 index. Therefore, although several studies concluded that different components of financial development may have distinct impacts on energy consumption based on the samples of specific countries [23,25], our empirical results indicate that both financial institutions and the financial market have positive effects on energy consumption from the worldwide perspective. These empirical results are similar with the works of Sadorsky [10], Sadorsky [16], and Al-mulali and Lee [17].

### 3.4. Empirical Analysis of the National Difference

The full-sample regressions provided us a comprehensive view of the effect of financial development on energy consumption. However, the regressions were unable to detect the potential discrepancy of this effect in different types of countries in distinct stages of development. Therefore, we divided our sample countries into two groups, developed and developing countries, to examine the national difference of the relationship of financial development and energy consumption in a unique framework. Table 8 presents the empirical results.

The coefficient of financial development for developed countries was statistically insignificant; conversely, the coefficient of financial development for developing countries was positive and significant at the 1% level. These empirical results indicated that financial development has no obvious impact on energy consumption in developed countries but has a positive effect in developing countries.

These findings suggest that the effect of financial development on energy consumption has a distinct national difference: For developed countries, the promoting effect of financial development on energy consumption is offset by its inhibitory effect. This signifies that although the financial development has no reduction effect, it would not increase the aggregate energy consumption. However, for developing countries, the promoting effect of financial development on energy consumption dramatically exceeds its inhibitory effect, having a significantly positive effect on energy consumption. This conclusion is consistent with the works of Ahmed [18], Farhani and Solarin [20], and Topcu and Payne [25].

Considering the national differences, policymakers should discreetly evaluate the impact of financial development on energy consumption for their own countries and coordinate the relationship on the basis of specific circumstances and endowments.

## 4. Robustness Checks

Next, we conducted two robustness checks to verify the reliability of the results. First, we adopted a different index to measure financial development; second, we expanded the sample periods.

### 4.1. Different Proxy Variables of Financial Development

We used the indexes of financial development proposed by Svirydzenka [36] as proxy variables of the overall financial development and its components due to their favorable characteristics. Then, we adopted another index, domestic credit to the private sector (% of GDP), to measure the financial development, which is also widely used in the literature [38,39,40], to verify the reliability of previous conclusions. Table 9 presents the empirical results.

Table 9 indicates that the coefficients of financial development are positive and significant for the full sample and developing countries, whereas the coefficient is insignificant for developed countries. These results suggest that the overall financial development has a positive impact on energy consumption for the full sample and developing countries, but no obvious effect for developed countries, which is consistent with the results of regressions using the FD1, FI, and FM indexes.

### 4.2. Longer Sample Periods

In the previous section, we chose a sample period of 24 years from 1991 to 2014, as this could provide a much larger sample size of up to 120 countries. Next, we analyzed the impact of financial development on energy consumption by extending the sample period to earlier decades to investigate the consistency of the previous conclusions in the long term. We extended the sample period, i.e., to 1981–2014, and conducted two regressions using the FD1 and FD2 indexes. Table 10 presents the empirical results.

Table 10 shows that the coefficients of FD1 and FD2 are all positive and significant at least at the 10% level, which is consistent with the previous conclusions, although these two regressions contained fewer sample countries. We also extended the sample period to much earlier decades, including 1970–2014 or 1960–2014; however, the lack of available country panel data was unable to support the empirical analysis.

## 5. Conclusions and Policy Implications

In this study, we investigated the influences of overall financial development and its components on energy consumption using the system GMM method and the panel data of 120 countries. We further analyzed national differences by dividing our sample into developed and developing countries. The empirical results indicated that financial development can increase energy consumption from the global perspective; its two components, the development of financial institutions and the financial market, have similar effects on energy consumption. The analysis of national differences proved that this effect only exists in developing countries; in developed countries, financial development has no obvious impact on energy consumption. Two robustness checks were conducted and verified the reliability of the results.

Based on the empirical results, we outline the following policy implications: (1) The development of the financial sector is widely thought to be beneficial for various aspects of the economy; however, financial development might not restrain the increase in energy consumption from the global perspective. Therefore, policymakers should discreetly analyze the impact of financial development on energy consumption on the basis of specific conditions and carefully examine their relationship in individual countries when formulating energy policies. (2) The analysis of national differences indicated that financial development has no obvious effect on energy consumption in developed countries, but significantly positively impacted energy consumption in developing countries. This suggests that although financial development was an important factor promoting the economic growth of developing countries, it could reduce energy security and environmental quality. Considering these features of financial development in developing countries, policymakers should emphasize the inhibitory effect of financial development, such as by encouraging the financial sector to provide more loans to high-tech enterprises to improve energy efficiency, which is conducive to realizing sustainable development.

Overall, there are two main limitations of our research: (1) Our empirical results indicated that an obvious national difference of the influence of the financial development on energy consumption existed between developed and developing countries, however we did not conduct further research concerning the reason of its appearance. (2) We analyzed from the worldwide perspective which could provide us a macro perception on this topic, therefore many micro factors are inevitably ignored. For instance, many economic or political events took place during the sample period which might have affected the relationship between financial development and energy consumption, which were not taken into consideration. This is because we prefered to concentrate our research objective on the worldwide perspective and, in fact, it is authentically difficult to solve too many issues in a single paper.

Considering the above limitations, we suggest focusing on the relationship between financial development and energy consumption from the nonlinear perspective in the future research. The inconsistent conclusions of the literature in this realm might be caused by one or more factors which could significantly affect the influence of financial development on energy consumption. In addition, it is also necessary to pay close attention to the micro events that occurred in recent decades relating to energy consumption (namely, the issue of structural break), as this could provide us a more specific view of the relationship between financial development and energy consumption.

## Figures and Tables

**Table 1:** Framework of the index proposed by Svirydzenka [36].

Comprehensive Index	First-Level Sub-Index	Second-Level Sub-Index
Financial Development (FD)	Financial Institution (FI)	Depth (FID)
		Access (FIA)
		Efficiency (FIE)
	Financial Market (FM)	Depth (FMD)
		Access (FMA)
		Efficiency (FME)

Source: International Monetary Fund (IMF) website [37].

**Table 2:** Variable descriptions.

Variable	Symbol	Measurable Indicator
Energy consumption	EC	Energy use (kg of oil equivalent per capita)
Financial development 1	FD1	A comprehensive index proposed by Svirydzenka [36]
Financial development 2	FD2	Domestic credit to the private sector (% of GDP)
Financial institution	FI	Index of financial institution proposed by Svirydzenka [36]
Financial market	FM	Index of financial market proposed by Svirydzenka [36]
Trade openness	TRADE	Total import and export (% of GDP)
Urbanization	URBAN	Urban population (% of total population)
Industrial structure	IND	Industrial value added (% of GDP)

Note: GDP, gross domestic product.

**Table 3:** Descriptive statistics of the main regression.

Variable	Obs.	Mean	SD	Min.	Max.
EC	2880	2234.595	2334.522	113.4227	18178.14
FD1	2880	0.339195	0.225983	0.019652	1
TRADE	2880	85.15676	54.69396	0.167418	442.62
URBAN	2880	60.39867	20.92772	9.18	100
IND	2880	29.57141	11.4977	2.525526	87.79689

Note: Obs., number of observations; SD, standard deviation.

**Table 4:** Results of panel unit root tests.

Variable	Breitung	Fisher	HT	IPS	LLC	Conclusion
EC	−0.4491 (0.3267)	−10.5772 *** (0.0000)	0.8525 *** (0.0079)	−2.6158 *** (0.0045)	−9.4737 *** (0.0000)	Stationary
FD1	−1.3563 * (0.0875)	−18.0671 *** (0.0000)	0.8042 *** (0.0000)	−5.1424 *** (0.0000)	−1.3699 * (0.0854)	Stationary
TRADE	−2.2328 ** (0.0128)	−13.8772 *** (0.0000)	0.6190 *** (0.0000)	−6.1242 *** (0.0000)	−5.1652 *** (0.0000)	Stationary
URBAN	−4.1324 *** (0.0000)	−8.9454 *** (0.0000)	0.9974 (0.3196)	−8.3033 *** (0.0000)	−4.6378 *** (0.0000)	Stationary
IND	−5.8071 *** (0.0000)	−16.1230 *** (0.0000)	0.9861 *** (0.0057)	−3.0726 *** (0.0011)	−4.4094 *** (0.0000)	Stationary

Notes: HT, Harris–Tzavalis test; IPS, Im–Pesaran–Shin test; LLC, Levin–Lin–Chu test. The values in parentheses are the *p*-values. ***, **, and * indicate significance at 1%, 5%, and 10% levels, respectively.

**Table 5:** Results of co-integration tests.

Method	Statistics
Kao	3.2201 *** (0.0006)
Pedroni	12.3390 *** (0.0000)
Wester Lund	−3.1519 *** (0.0008)

Notes: Values in parentheses are the *p*-values. *** indicates significance at 1% level.

**Table 6:** Results of the full sample regressions with FD1.

Variable	Full Sample with FD1			
L.EC	0.9669 *** (0.0130)	0.9657 *** (0.0136)	0.9575 *** (0.0166)	0.9452 *** (0.0167)
FD1	0.1058 ** (0.0447)	0.1084 ** (0.0455)	0.0797 ** (0.0322)	0.1218 *** (0.0344)
TRADE	-	0.0023 (0.0046)	−0.0020 (0.0051)	−0.0017 (0.0051)
URBAN	-	-	0.1029 ** (0.0469)	0.1127 ** (0.0454)
IND	-	-	-	0.1256 *** (0.0325)
Constant	0.2084 *** (0.0782)	0.2146 *** (0.0804)	0.2248 *** (0.0810)	0.2560 *** (0.0774)
Number of countries	120	120	120	120
AR (1)	−6.12 *** (0.000)	−6.12 *** (0.000)	−6.10 *** (0.000)	−6.09 *** (0.000)
AR (2)	0.45 (0.655)	0.44 (0.659)	0.44 (0.663)	0.42 (0.674)
Hansen test	112.46 (0.246)	112.32 (0.249)	112.46 (0.246)	111.68 (0.263)

Note: L.EC, first-order lag term of energy consumption; AR (1) and AR (2), the first-order and second-order autocorrelation estimators, respectively. Values in parentheses under the coefficients are the standard errors, and the values in parentheses under the statistics of AR (1), AR (2), and the Hansen test are the *p*-values. ***, and ** indicate significance at 1%, and 5% levels, respectively. The following tables have the same notes as in this table; to avoid duplication and save space, these notes are no longer presented.

**Table 7:** Results of the full sample regressions with FI and FM.

Variable	Financial Institution	Financial Market
L.EC	0.9701 *** (0.0108)	0.9668 *** (0.0116)
FI/FM	0.0706 *** (0.0257)	0.0463 *** (0.0168)
TRADE	−0.0016 (0.0031)	0.0005 (0.0043)
URBAN	0.0487 * (0.0271)	0.0792 ** (0.0358)
IND	0.0966 *** (0.0257)	0.0723 *** (0.0236)
Constant	0.1369 *** (0.0470)	0.1659 *** (0.0555)
Number of countries	119	117
AR (1)	−6.80 *** (0.000)	−6.69 *** (0.000)
AR (2)	0.22 (0.828)	0.60 (0.549)
Hansen test	100.00 (0.112)	98.41 (0.135)

***, **, and * indicate significance at 1%, 5%, and 10% levels, respectively.

**Table 8:** Results of sub-sample regressions.

Variable	Developed Countries	Developing Countries
L.EC	0.9826 *** (0.0455)	0.8892 *** (0.0364)
FD1	0.0199 (0.0246)	0.2461 *** (0.0776)
TRADE	−0.0040 (0.0048)	0.0268 (0.0188)
URBAN	0.0522 (0.0641)	0.2491 ** (0.0978)
IND	0.1176 (0.0970)	0.1800 ** (0.0711)
Constant	0.0656 (0.3000)	0.4916 *** (0.1712)
No. countries	34	86
AR (1)	−3.15 *** (0.002)	−5.13 *** (0.000)
AR (2)	1.54 (0.125)	−0.42 (0.674)
Hansen test	32.74 (0.206)	74.49 (0.174)

***, and ** indicate significance at 1%, and 5% levels, respectively.

**Table 9:** Results of the full sample and sub-sample regressions with FD2.

Variable	Full Sample	Developed Countries	Developing Countries
L.EC	0.9557 *** (0.0169)	0.9725 *** (0.0374)	0.8853 *** (0.0392)
FD2	0.0320 *** (0.0119)	0.0011 (0.0063)	0.0879 *** (0.0271)
TRADE	−0.0023 (0.0054)	−0.0061 (0.0055)	0.0125 (0.0186)
URBAN	0.1196 ** (0.0558)	0.0787 (0.0536)	0.2907 *** (0.1110)
IND	0.0939 *** (0.0281)	0.1389 (0.0885)	0.1987 *** (0.0753)
Constant	0.2111 *** (0.0790)	0.1365 (0.2599)	0.5322 *** (0.1902)
Number of countries	120	34	86
AR (1)	−6.08 *** (0.000)	−3.15 *** (0.002)	−5.06 *** (0.000)
AR (2)	0.41 (0.683)	1.53 (0.126)	−0.46 (0.643)
Hansen test	113.85 (0.219)	31.17 (0.184)	73.65 (0.192)

***, and ** indicate significance at 1%, and 5% levels, respectively.

**Table 10:** Results of the full sample regressions with the longer period.

Variable	FD1	FD2
L.EC	0.9755 *** (0.0186)	0.8620 *** (0.0439)
FD1/FD2	0.0862 * (0.0510)	0.1278 *** (0.0454)
TRADE	0.0001 (0.0045)	−0.0156 (0.0176)
URBAN	0.0271 (0.0389)	0.3675 *** (0.1099)
IND	0.1199 *** (0.0400)	0.2370 ** (0.1186)
Constant	0.1043 (0.0842)	0.6318 *** (0.2232)
Number of countries	61	55
AR (1)	−4.07 *** (0.000)	−3.83 *** (0.000)
AR (2)	1.16 (0.247)	1.15 (0.249)
Hansen test	58.14 (0.650)	52.30 (0.852)

***, ** and * indicate significance at 1%, 5% and 10% levels, respectively.

## Appendix A. Sample Countries and Classification

**Table A1:** Sample countries and classification.

**Developed Countries**				
Australia	Austria	Belgium	Cyprus	Czech Republic
Denmark	Estonia	Finland	France	Germany
Greece	Hong Kong SAR, China	Iceland	Ireland	Israel
Italy	Japan	Korea, Republic	Latvia	Lithuania
Luxembourg	Malta	Netherlands	New Zealand	Norway
Portugal	Singapore	Slovak Republic	Slovenia	Spain
Sweden	Switzerland	United Kingdom	United States	
**Emerging Market and Developing Countries**				
Albania	Algeria	Angola	Argentina	Azerbaijan
Bahrain	Bangladesh	Belarus	Benin	Bolivia
Bosnia and Herzegovina	Botswana	Brazil	Brunei Darussalam	Bulgaria
Cambodia	Cameroon	Chile	China	Colombia
Congo, Democratic Republic	Congo, Republic	Costa Rica	Cote d’Ivoire	Croatia
Dominican Republic	Ecuador	Egypt, Arab Republic	El Salvador	Gabon
Georgia	Ghana	Guatemala	Haiti	Honduras
Hungary	India	Indonesia	Iran, Islamic Republic	Jamaica
Jordan	Kazakhstan	Kenya	Kyrgyz Republic	Lebanon
Libya	Macedonia, FYR	Malaysia	Mauritius	Mexico
Moldova	Mongolia	Morocco	Mozambique	Myanmar
Namibia	Nepal	Nicaragua	Niger	Nigeria
Oman	Pakistan	Panama	Paraguay	Peru
Philippines	Poland	Romania	Russian Federation	Saudi Arabia
Senegal	South Africa	Sri Lanka	Sudan	Tajikistan
Tanzania	Thailand	Togo	Tunisia	Turkey
Ukraine	United Arab Emirates	Uruguay	Venezuela, RB	Vietnam
Zambia				
